# Self-excision of the BAC sequences from the recombinant Marek's disease virus genome increases replication and pathogenicity

**DOI:** 10.1186/1743-422X-5-19

**Published:** 2008-01-30

**Authors:** Yuguang Zhao, Lawrence Petherbridge, Lorraine P Smith, Sue Baigent, Venugopal Nair

**Affiliations:** 1 Viral Oncogenesis Group, Institute for Animal Health, Compton, Berkshire RG20 7NN, UK

## Abstract

Cloning of full length genomes of herpesviruses as bacterial artificial chromosomes (BAC) has greatly facilitated the manipulation of the genomes of several herpesviruses to identify the pathogenic determinants. We have previously reported the construction of the BAC clone (pRB-1B5) of the highly oncogenic Marek's disease virus (MDV) strain RB-1B, which has proven to be a valuable resource for elucidating several oncogenic determinants. Despite the retention of the BAC replicon within the genome, the reconstituted virus was able to induce tumours in susceptible chickens. Nevertheless, it was unclear whether the presence of the BAC influenced the full oncogenic potential of the reconstituted virus. To maximize the closeness of BAC-derived virus to the parental RB-1B strain, we modified the existing pRB-1B5 clone by restoring the Us2 and by introducing SV40-*cre *cassette within the *lox*P sites of the mini-F plasmid, to allow self-excision of the plasmid sequences in chicken cells. The reconstituted virus from the modified clone showed significant improvement in replication *in vitro *and *in vivo*. Excision of the BAC sequences also enhanced the pathogenicity to levels similar to that of the parental virus, as the cumulative incidence of Marek's disease in groups infected with the recombinant and the parental viruses showed no significant differences. Thus, we have been able to make significant improvements to the existing BAC clone of this highly oncogenic virus which would certainly increase its usefulness as a valuable tool for studies on identifying the oncogenic determinants of this major avian pathogen.

## Background

Marek's disease virus (MDV) is one of the most contagious and highly oncogenic alphaherpesvirus that induces T-cell lymphomas in the chickens [1,2]. Apart from the economic significance to the poultry industry with annual losses ranging between US$ 1–2 billion [3], MD is also a valuable model for studying the principles of virus-induced oncogenesis [4,5]. Studies on understanding the role of viral genes in the biology of MDV have been greatly facilitated by the construction of the bacterial artificial chromosome (BAC) clones of MDV [6,7]. The ability for rapid manipulation of BAC clones using well-established mutagenesis techniques in *E. coli *[8,9] and easy reconstitution of mutant viruses in transfected chicken cells has made this technique a valuable and efficient tool for studying MDV gene functions. We have previously reported the construction of pRB-1B-5, a BAC clone generated from the highly oncogenic RB-1B strain [10] of MDV by inserting the mini-F plasmid into the non-essential Us2 region of the genome [11]. Retention of oncogenicity of MDV (vRB-1B5) reconstituted from this clone has enabled the use of this clone in various studies to examine the oncogenic determinants using natural *in vivo *models of MD [5,12-15].

Although vRB-1B5 was capable of inducing tumours in susceptible chickens, the parental RB-1B virus appeared to show higher oncogenicity than the recombinant vRB-1B5 measured by the time of onset and incidence of tumours [this study and [12]]. As vRB-1B5 carried the mini-F plasmid sequences in the Us2 locus, we wanted to examine whether the reduced oncogenic property of vRB-1B5 is related to the presence of the extra foreign sequences. For this, we sought to modify the pRB-1B5 clone by introducing a *cre-lox *site-specific recombination system to excise the mini-F plasmid exploiting the flanking *Lox*P sites in pRB-1B5. Since the eukaryotic SV40 promoter-driven *cre*-expression cassette is non-functional in prokaryotes, this method would not affect the replication of the BAC DNA in bacteria. Additionally, the inclusion of an intron sequence within the *cre *gene further guarantees that no functional *cre *is expressed in bacteria [16]. Once the MDV BAC DNA is transfected into chicken cells, the eukaryotic SV40 promoter becomes functional and the splicing of the intron leads to the expression of *cre*, which cleaves at the two *lox*P sites that flank the BAC backbone cassette, including the *cre *gene itself. This results in the excision of the mini-F plasmid leaving just one copy of *lox*P site in the virus genome. The *cre/lox*P system has been used on BAC clones of other herpesviruses such as pseudorabies virus [16], human cytomegalovirus [17] and rhesus cytomegalovirus [18] to generate self-excisable viruses. In this study, we examined whether the self-excised MDV generated from the modified pRB-1B5 has increased viral replication and pathogenesis in a natural infection model in susceptible chickens.

## Findings

**Figure 1:**
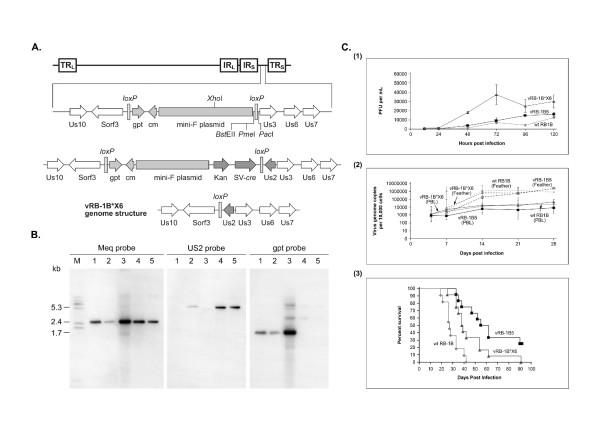
**A**: Schematic diagram showing the construction of the self excisable pRB-1B*6X clone. Top – Genome structure of MDV showing the expanded Us2 locus: Us10, Sorf3, *gpt *(guanine phosphoribosyl transferase gene), CM (chloramphenicol resistance gene), mini-F plasmid, flanking loxP sites, Us3, Us6 and Us7, as well as the unique restriction sites are shown. Middle – Genome structure of pRB-1B*6X clone showing the restored Us2, the inserted *Kan *flanked by FRT sites and the SV-*cre *cassette inside the *lox*P region. Bottom – Genome structure of vRB-1B*6X virus after the excision of the SV-*cre *and the *Kan *cassette in the CEF. **B**: Southern blotting hybridization of *Eco*RI-digested DNA prepared from either the BAC clone or CEF infected with wild type or the recombinant viruses, hybridized, stripped and probed sequentially with DIG-labelled MEQ, Us2 or *gpt *probes. M – Molecular weight markers showing the size of MEQ (2.4 kb), Us2 (5.3 kb) and *gpt *(1.7 kb) fragments. Lane 1 – pRB-1B5 DNA; Lane 2 – pRB-1B*6X DNA; Lane 3 – vRB-1B5-infected CEF DNA; Lane 4 – vRB-1B*6X-infected CEF DNA; Lane 5 – wtRB-1B-infected CEF DNA. **C**: Comparison of *in vitro *and *in vivo *replication and percentage survival of birds infected with RB-1B viruses (**1**) *in vitro *replication kinetics of vRB-1B5 (black square), vRB-1B*6X (black triangle) and wtRB-1B (grey diamond) viruses in CEF calculated as PFU per mL at different time points after infection (**2**) kinetics of *in vivo *replication of vRB-1B5 (black square), vRB-1B*6X (black triangle) and wtRB-1B (grey diamond) viruses determined from the viral genome copy numbers in PBL (continuous line) and feather DNA (dotted line) using TaqMan real-time qPCR of meq quantitative PCR. (**3**) Comparison of the cumulative mortality rates at different time points after infection with vRB-1B5 (black square), vRB-1B*6X (black triangle) and wtRB-1B (grey diamond) viruses. The survival rates showed statistically significant differences between the wtRB-1B and vRB-1B5 (p =< 0.0001) and wtRB-1B virus and vRB-1B*X6 groups (p = 0.0064).

The modification of pRB-1B was carried out as shown in Fig 1A. Briefly, the Us2-Us3 sequence was amplified from the wtRB-1B virus-infected chicken embryo fibroblasts (CEF) genomic DNA by PCR using primers Us2F (5'-GTTAATTAACGACAGACCTACTTGCTACCA) and Us3R (5'-CTCGAGGTATGGCCATGTGGTCTCTA) that contained the *Pac*I and *Xho*I restriction sites respectively. The *Xho*I-*Pac*I fragment containing partial mini-F sequence released from the plasmid pDS-HA1 [8] was linked to the above Us2-Us3 fragment through *Pac*I-*Sal*I site. The SV40-*cre *fragment released from pYD-C66 (kindly provided by Thomas Shenk, Princeton, USA) as *EcoR*I-*Xho*I fragment was blunt-ended and inserted into *Pme*I site of the above plasmid. Finally a blunt ended FRT-Kan cassette from pKD13 was further inserted into *BstE*II site (blunt ended) of the above plasmid. The resulting construct pPartial-F-Kan-*cre*-Us2-Us3 was used for making self-excisable BAC clone of RB-1B virus by lambda Red mutagenesis [19] by co-transforming with pRB-1B5 in bacterial strain EL250 and selecting in Luria-Bertani (LB) plates containing chloramphenicol (30 μg/ml) and kanamycin (50 μg/ml) at 32°C for 24–36 hours. Targeted recombination in the kanamycin and chloramphenicol-resistant colonies was confirmed by the detection of a 3.5-kb product by PCR using primer pairs Us2-F (5'-GGAATACATTCGAGCGCAA) & Us7-R (5'-CTATAGACCAGATGCCTCGAA) located in the Us2 and Us7 respectively. The *Kan *cassette flanked by the FRT sequences was flipped off by adding 0.2% L-arabinose for 1 hour and selecting on chloramphenicol-containing LB plates. DNA extracted from one of the chloramphenicol-resistant clones (pRB-1B*X6) was checked for infectivity by transfection into primary CEF using Lipofectamine (Invitrogen, Paisley, United Kingdom). The virus reconstituted virus from this clone, designated vRB-1B*X6, was grown up on CEF, titrated and stored in liquid nitrogen.

The integrity of the pRB-1B*X6 clone was initially checked by *Eco*RI restriction digestion (not shown) followed by Southern blot analysis using digoxigenin (DIG)-labelled DNA probes (Roche Applied Sciences, Hertfordshire, United Kingdom) using procedures described before [11]. The membrane was sequentially hybridized with probes specific for MEQ, Us2 region and the *E. coli *guanine phosphoribosyl transferase (*gpt*) sequence in the pDS-PHAI [8]. As expected, a single 2.4 kb MEQ specific band was present in all lanes including the DNA from pRB-1B5, pRB-1B*X6, vRB-1B5, vRB-1B*X6, as well as the wtRB-1B virus (Fig. 1B). Stripping and reprobing the membrane with the Us2 probe showed no signals pRB1B-5 and vRB-1B5 (lanes 1 and 3), confirming the deletion of the Us2 gene in these constructs [11]. Detection of the Us2-specific band of the similar size in the wtRB-1B (lane 5) both in pRB-1B*X6 (lane 2) and vRB-1B*X6 (lane 4) confirmed the repair of the Us2 deletion in this construct. When the same membrane was further stripped and probed with the *gpt *probe, specific signals were detected in pRB-1B5 (lane 1), pRB-1B*X6 (lane 2) and vRB-1B5 (lane 3), demonstrating the presence of the mini-F plasmid in these DNA samples. The absence of *gpt *signals in the DNA sample from vRB-1B*X6 (lane 4), similar to the wtRB-1B virus (lane 5), confirms the self-excision of the mini-F plasmid during the growth of the virus in the CEF, following the expression of the functional *cre *in these cells. Thus the data from the Southern blot hybridization showing the presence of MEQ and Us2 and absence of mini-F plasmid confirmed that the genome structure of the vRB-1B*X6 is similar to the wtRB-1B virus. We also examined the status of expression of Us2 by reverse transcription PCR in the cells infected with the 3 viruses. As expected, both vRB1B-X6 and wtRB-1B virus-infected cells amplified a PCR product of the expected size, cells infected with vRB1B-5 virus was negative (data not shown).

In order to examine whether the above modifications have given any growth advantage to the vRB-1B*X6 over vRB-1B5, we first compared its *in vitro *growth of the two viruses in CEF. As shown in the Fig 1C (1), at all the time points after 24 hours post infection vRB-1B*X6 showed higher titres than vRB-1B5, demonstrating that the modification did have a positive effect on virus replication *in vitro*. The wtRB-1B virus, less adapted to replicate in CEF, consistently showed lower *in vitro *levels than the other viruses. We then asked whether the increased *in vitro *replication of the vRB-1B*X6 is also reflected in the replication *in vivo*. For this, we monitored the MDV genome copy numbers in the peripheral blood leukocytes (PBL) of 5 line P SPF (specific-pathogen-free) chickens infected with 1000 p. f. u. of vRB-1B5 and vRB-1B*X6 viruses at different days up to 28 days post infection using real-time quantitative PCR [20]. MDV genome copy numbers of both viruses reached the plateau at 14 days post infection, after which the titres were maintained. The genome copy numbers of vRB-1B*X6 virus was at a higher level than that of vRB-1B5 at all time points demonstrating higher *in vivo *replication in PBL. In this respect, vRB-1B*X6 virus showed a replication kinetics almost identical to the wtRB-1B virus [Fig 1C (2)]. The differences in the replication trend between the 3 viruses in the PBL was also reflected in the feather DNA samples [Fig 1C (2)].

We then asked whether the increased replication ability of vRB-1B*X6 is associated with higher pathogenicity in an infection model. For this, groups (n = 10) of one-day old MD-susceptible specific-pathogen-free line P (B^19^/B^19^) chickens lacking maternal antibodies to MDV were infected intra-abdominally with 1000 p. f. u. of vRB-1B5, vRB-1B*X6 and wtRB-1B viruses. All procedures on experimental birds were approved by the Institute for Animal Health Ethical Committee and carried out in accordance with the Project Licence 30/2145 issued by the United Kingdom Home Office. All the groups of infected birds, together with a group of uninfected control birds, were maintained in isolation and observed for 90 days. Cumulative occurrence of MD for the different groups, based on the incidence gross or histological lesions, was used to calculate the survival rates, and the statistical differences between the different groups were calculated using Kaplin-Meier log rank test for survival [21]. The survival curves showed significant increase in the median time to death between the birds infected with vRB-1B*X6 and vRB-1B5 viruses [Fig 1C (3)]. By including the wtRB-1B virus-infected group in the experiment, we were able to examine the pathogenicity of the two recombinant cloned viruses with that of the wtRB-1B virus stock. There were significant differences in the survival rates between birds infected with wtRB-1B virus and vRB-1B5 viruses (p =< 0.0001), as well as between the birds infected with wtRB-1B virus and vRB-1B*X6 viruses (p = 0.0064). Thus our studies demonstrate that the excision of the mini-F plasmid can generate recombinant viruses with increased pathogenicity. These observations were supported in a recent study where the excision of the mini-F sequences was achieved using a different strategy [22]. Thus, we have been able to achieve a significant improvement to the existing BAC clone of this highly oncogenic virus to generate virus stocks with very close biological properties as the parent wtRB-1B virus. This would certainly increase its usefulness as a valuable tool for studies on genome manipulation to identify the oncogenic determinants of this major avian pathogen.

## Competing interests

The author(s) declare that they have no competing interests.

## Authors' contributions

YZ contributed to design, perform the experiment and draft the manuscript. LP participated in the experiment. LPS and SB conducted animal experiments and quantitative PCR analysis. VN supervised the study and drafted the manuscript.
